# On the specific heat capacity enhancement in nanofluids

**DOI:** 10.1186/s11671-015-1188-5

**Published:** 2016-02-13

**Authors:** Reinhard Hentschke

**Affiliations:** Fachbereich Mathematik und Naturwissenschaften, Bergische Universität, Wuppertal, D-42097 Germany

**Keywords:** Energy storage, Solar power plants, Heat transfer nanofluids, Specific heat capacity

## Abstract

Molten salts are used as heat transfer fluids and for short-term heat energy storage in solar power plants. Experiments show that the specific heat capacity of the base salt may be significantly enhanced by adding small amounts of certain nanoparticles. This effect, which is technically interesting and economically important, is not yet understood. This paper presents a critical discussion of the existing attendant experimental literature and the phenomenological models put forward thus far. A common assumption, the existence of nanolayers surrounding the nanoparticles, which are thought to be the source of, in some cases, the large increase of a nanofluid’s specific heat capacity is criticized and a different model is proposed. The model assumes that the influence of the nanoparticles in the surrounding liquid is of long range. The attendant long-range interfacial layers may interact with each other upon increase of nanoparticle concentration. This can explain the specific heat maximum observed by different groups, for which no other theoretical explanation appears to exist.

## Background

A standard problem in courses on Statistical Mechanics, when the topic is Stefan’s law, is the calculation of the size of a “solar panel” large enough to collect the energy currently consumed by the earth’s population. Even though this is an academic problem, the resulting area is surprisingly small, providing a feeling for the vast amount of solar energy received by the surface of the earth. One of the technologies, being developed in this context, is that of thermal solar power plants, especially of the parabolic trough and power tower types. Inside these plants, heat transfer fluids, e.g., molten salts, are used for energy transport as well as storage [1]. Heat transfer fluids must meet certain key criteria - aside from being inexpensive. They should be thermally stable at high temperatures. Their melting point should be as low as possible. The same is true for their vapor pressure at high temperatures (several hundred degree Celsius). Other aspects include corrosivity, viscosity, thermal conductivity, toxicity, etc.

The thermodynamic property of interest in this study is the specific heat capacity, i.e., the heat capacity in units of joules per gram Kelvin, of the heat transfer fluid, which should be as high as possible. The aforementioned molten salts or rather salt mixtures do possess a wide temperature range of useful application - an advantage outweighing their comparatively low specific heat capacity of about 0.75 to 2 J/(gK). However, a number of experiments (cf. the recent review [2]) do show that the specific heat capacity of the base salt mixture, here and in the following we talk about *c*_*P*_, the specific heat capacity at constant pressure, is substantially enhanced by addition of nanoparticles in small amounts (≈ 1 wt.%), i.e., particles whose size distribution is peaked around 10 to 100 nm. Salt mixtures doped with nanoparticles belong to a larger and more general class of fluids, so-called nanofluids.

Nanofluids doped with suspended nanoparticles do show enhanced thermal conductivities [3–8]. The effect of nanoparticle addition on the specific heat capacity of the fluids, however, does not yield a consistent picture (e.g., [9–37]). Das and co-workers [9–11] find reduced specific heat capacities of nanofluids consisting of silicon dioxide, zinc oxide, and alumina nanoparticles, respectively, dispersed in a mixture of water and ethylene glycol compared to the base fluid. The specific heat capacity of the nanofluid is found to decrease with increasing nanoparticle concentration. Zhou and Ni [12] also find a reduced specific heat capacity of the water-based alumina nanofluid and a similar decrease of specific heat capacity with increasing particle concentration. An extensive study by O’Hanley et al. [13] confirms this detrimental effect of various types of nanoparticles on the heat capacity of water. Recent work by Sekhar and Sharma [14] comes to similar conclusions. In contrast, Nelson et al. [15] find that *c*_*P*_ of polyalphaolefin is enhanced by 50 % when mixed with graphite nanoparticles at 0.6 % mass fraction. The *c*_*P*_ of Li _2_CO_3_/K_2_CO_3_ eutectic salt is enhanced by 19 % when mixed with carbon nanotubes at 1 % mass fraction as reported by Shin et al. [16]. Refs. [17, 18] by Jo and Banerjee are continuations of this work using the same base fluid. In [17], the effect of nanoparticle dispersion is studied based on graphite nanoparticles. Greater enhancement in the specific heat capacity was observed from the nanomaterial samples with more homogeneous dispersion of the nanoparticles. In [18], multiwalled CNTs (carbon nanotubes) were dispersed using a surfactant (SDS) to obtain homogeneous dispersion. Four different concentrations (0.1, 0.5, 1, and 5 wt.%) of CNT were employed. It was observed that the specific heat capacity is enhanced by doping with the nanotubes in both solid and liquid phase. In addition, the enhancements of the specific heat capacity are increased with increase of the CNT concentration. Zhou et al. [19] find a maximum enhancement of about 6 % of the specific heat capacity of their ethylene glycol-based CuO nanofluid. In addition, Shin and Banerjee [20–22] obtain specific heat capacity enhancements of about 14 and 19 % to 24 % in different nanofluids, i.e., Li _2_CO_3_/K_2_CO_3_ eutectic and chloride eutectic, doped with 1 wt.% SiO _2_ nanoparticles. Most recently, they report specific heat capacities in the Li _2_CO_3_/K_2_CO_3_ (62:38) eutectic doped with 1.5 wt.% silica nanoparticles (size between 2 and 20 nm), which are about 120 % above the base salt specific heat capacity [23]. These researchers have also used alumina nanoparticles in again the same base salt mixture [24]. They report an enhancement of around 30 % at 1 % mass concentration. Lu and Huang [25] study the NaNO _3_/KNO _3_ (60:40) liquid base salt mixture doped with alumina nanoparticles of two distinct sizes. Their key observation is that *c*_*P*_ is larger for the nanofluid containing the larger nanoparticles under otherwise identical conditions. This is also found by Dudda and Shin [26] in NaNO _3_/KNO _3_ (60:40) doped (1 wt.%) with SiO _2_ nanoparticles. In contrast, Tiznobaik and Shin [27] do find no influence of particle size in the Li _2_CO_3_/K_2_CO_3_ eutectic doped with silicon dioxide nanoparticles (5, 10, 30, and 60 nm in diameter - same sizes as in [26]). In another publication, Tiznobaik and Shin [28] tie the enhancement of the specific heat capacity observed in the previous system to the formation of nanostructures surrounding the particles. In subsequent work Shin, Tiznobaik, and Banerjee [29] discuss the possible role of fractal flocs formed by the nanoparticles on the enhancement of the specific heat capacity in nanofluid. The two investigations by Chieruzzi [30] and Lasfargues et al. [31, 32] study the eutectic mixture of NaNO _3_/KNO _3_ doped with different nanoparticles at different concentrations. These researchers find that in general the specific heat capacity is enhanced by a few percent (up to about 10 % using copper oxide in [31]). Most notably, however, a maximum enhancement is observed in the range between 0.1 and 1 wt.% nanoparticle. An analogous maximum was also observed by Heilmann [33] using the same base salt doped with Al _2_O_3_ nanoparticles. Another confirmation of the maximum comes from Andreu-Cabedo [34]. They study the eutectic mixture of NaNO _3_/KNO _3_ doped with silica nanoparticles in the concentration range from 0.5 to 2 wt.%. Their maximum heat capacity increase is 25 %.

It is important to stress that the observation of enhanced heat capacity is not limited to nanofluids whose base is a binary mixture. Recently, Chieruzzi et al. [35] have studied a single component base fluid, KNO _3_, doped with different size nanoparticles of silica, alumina, and a mix thereof. In both the solid and the liquid phase, the authors do observe an increase of *c*_*P*_. At 1 wt.% added nanoparticles *c*_*P*_ in the solid phase is increased between 5 and 10 %. In the liquid phase, the increase is around 6 %. Ho and Pan [36] have looked for the optimal concentration of alumina nanoparticles in a molten ternary salt mixture. They obtain a specific heat capacity increase of about 20 % at a very small nanoparticle concentration, i.e., 0.063 wt.%. A still larger increase of *c*_*P*_ has been found by Paul [37], who has studied different nanoparticle-enhanced ionic liquids. Ionic liquids are salts whose cations are large organic species and anions are organic or inorganic species. Interestingly, the considerable increases of *c*_*P*_ reported in this work appear to be monotonous functions of nanoparticle volume fraction in the entire range of volume fractions studied (up to 2.5 wt.%).

In summary, it is probably safe to say that the specific heat capacity of salt mixtures is enhanced by every type of nanoparticle used. The particle mass concentration should not exceed 1 wt.%, however. Details of the methods of preparation may vary (e.g., the temperatures during drying of the samples). Quite independent of these variations, *c*_*P*_ enhancements are between 10 and 30 % on average. Doping water with nanoparticles thus far yields a decrease of the heat capacity. A molecular theory explaining the above effects of nanoparticles on the specific heat capacity of heat transfer fluids does not yet exist.

## Results and Discussion

The first part of this section, phenomenological heat capacity of nanofluids - the nanolayer concept, is an exposition of the existing phenomenological theory and where it fails to describe the experimental findings. The second part, phenomenological heat capacity of nanofluids - the mesolayer concept, presents arguments in support of the idea that the effect of the nanoparticles on the base fluid should be of much longer range than assumed previously. In the third part, the interacting mesolayer model, it is shown how this concept can be developed into a model explaining the specific heat maximum observed in several experimental studies. The section concludes with a suggestion on how to tie this phenomenological theory to molecular interaction.

*Phenomenological heat capacity of nanofluids - the nanolayer concept*: In certain simple cases, the dependence of the specific heat capacity of the nanofluid is well described by

(1) $$ c_{P}= \frac{M_{\text{liq}} c_{P,\text{liq}} +M_{\text{np}} c_{P,\text{np}}}{M_{\text{liq}}+M_{\text{np}}}.  $$

**Fig. 1:**
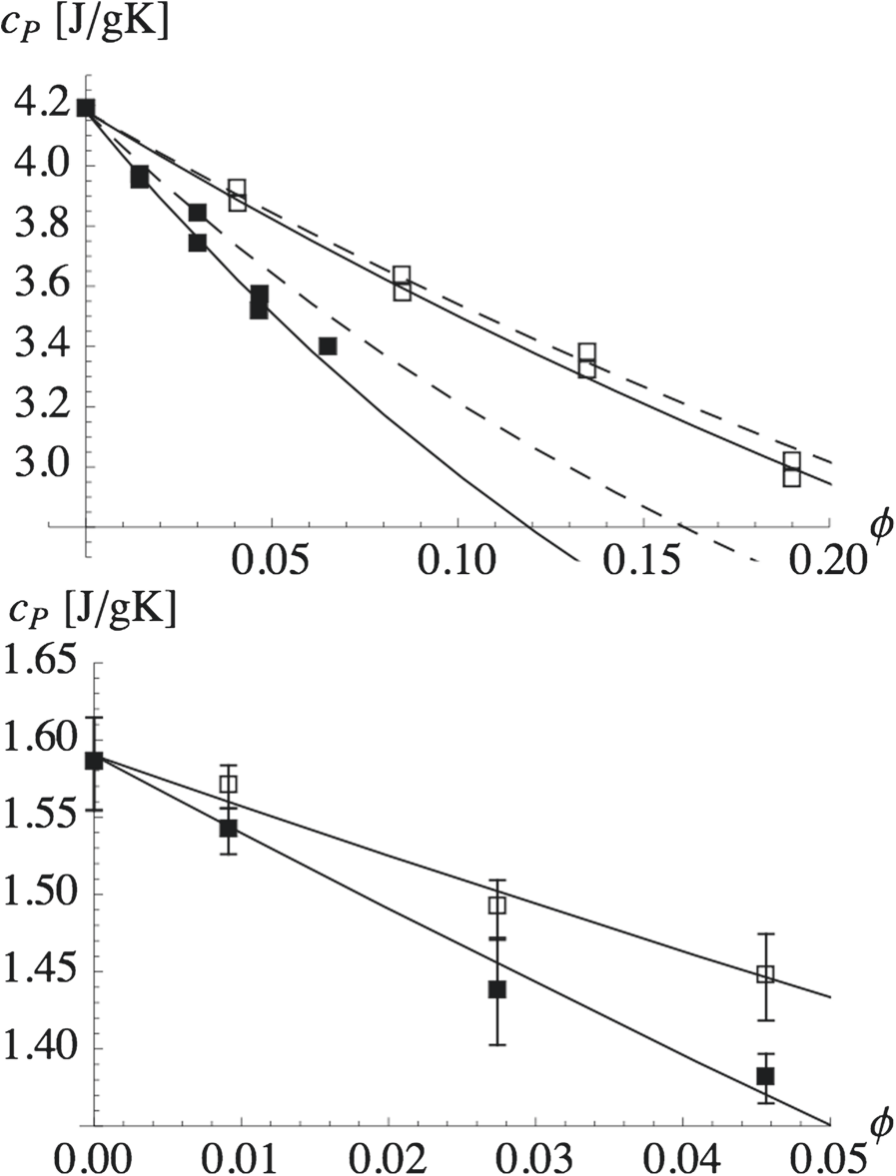
Specific heat capacity of the nanofluid vs. particle volume fraction. *Top*: Specific heat capacity of water-based nanofluids. Experimental data are taken from Figs. 2 and 8 in [13] (*open symbols*: silica; *closed symbols*: alumina). The temperature is 45 °C. *Dashed lines*: Eq. 1. *Solid lines*: Eq. 5 using *λ*=1.05 (silica) and *λ*=1.2 (alumina). *Bottom*: Specific heat capacities for NaNO _3_/KNO _3_ (60:40) doped with alumina nanoparticles. *Symbols*: data from Figure 5 in [25]. Two different size nanoparticles are used: 13 nm (*solid symbols*) and 90 nm (*hollow symbols*). *Solid lines*: Eq. 5 using *λ*=2.2 (13 nm particle size) and *λ*=1.7 (90 nm particle size). The other quantities needed to evaluate Eq. 5 were taken from [25]

Here *M*_liq_ is the mass of the base liquid, whereas *M*_np_ is the total mass of the nanoparticles. *c*_*P*,liq_ and *c*_*P*,np_ are the respective specific heat capacities of the two components. More precisely speaking, there are more than two components present if the base liquid is a mixture. The dashed lines in the top panel of Fig. 1 show this equation in comparison to experimental data from [13]. Notice that the volume fraction in the figure refers to volume fraction nanoparticles, *ϕ*, related to the mass ratio *y*=*M*_np_/*M*_liq_ via

(2) $$ \phi = \frac{y/\rho_{\text{np}}}{y/\rho_{\text{np}} + 1/\rho_{\text{liq}}} \quad \text{or} \quad y =\frac{\rho_{\text{np}}}{\rho_{\text{liq}}}\frac{\phi}{1-\phi} \;,  $$

where *ρ*_liq_ and *ρ*_np_ are the mass densities of the base liquid and the nanoparticles, respectively. In [13], the authors investigate water-based silica, alumina, and copper oxide nanofluids. In some cases, Eq. 1, e.g., the open symbols in the top panel of Fig. 1, provides a good description of the experimental data. In other cases, e.g., the closed symbols in the top panel of Fig. 1, Eq. 1 is off.

It is worth noting that the specific heat capacity of the nanoparticles, *c*_*P*,np_, is significantly below the specific heat capacity of the base fluid, *c*_*P*,liq_ - and not only in the case of water. Theoretical calculations of *c*_*P*,np_ indicate that the nanoparticles possess somewhat larger specific heat capacities (at high temperatures) than their bulk materials [38, 39]. However, the difference does not alter this statement. In particular, the concentration of nanoparticles in the experiments is so small that the difference between the nanoparticle’s *c*_*P*,np_ and the corresponding bulk-specific heat capacities is negligible in the present context.

Equation 1 may be improved by addition of an interface or nanolayer separating the bulk base fluid from the nanoparticle’s surface as has been done in [25] (Eq. 2) (cf. also [26] (Eq. 2)). It is assumed that this interface possess a characteristic thickness of between a couple to 10 nm. This interface contribution is introduced into the numerator of Eq. 1, i.e.

(3) $$ c_{P}= \frac{(M_{\text{liq}} -M_{i}) c_{P,\text{liq}} +M_{i} c_{P,i}+ M_{\text{np}} c_{P,\text{np}}}{M_{\text{liq}}+M_{\text{np}}}.  $$

Here, *M*_*i*_ is the mass of the base liquid in the interface around the nanoparticles, and *c*_*P*,*i*_ is the specific heat capacity associated with the interface. In addition, we define

(4) $$ \kappa = \frac{c_{P,i}}{c_{P,\mathrm{ liq}}}.  $$

*κ* is a factor by which the specific heat capacity in the interface differs on average from the specific heat capacity in the pure bulk fluid. Thus, Eq. 3 becomes

(5) $$ c_{P}= c_{P,\text{liq}} + x_{\text{np}} \left(c_{P,\text{np}} - \lambda c_{P,\text{liq}} \right),  $$

with

(6) $$ \lambda =1+(1-\kappa) \frac{M_{i}}{M_{\text{np}}}\;.   $$

The quantity *x*_np_=1−*x*_liq_=*M*_np_/(*M*_np_+*M*_liq_) is the mass fraction nanoparticles (notice: *x*_np_=*y*/(1+*y*)). In the limit *λ*=1 (or *κ*=1), this equation is identical to Eq. 1. However, if momentarily we treat *λ* as an adjustable parameter, we can improve the approximation to the data in Fig. 1 (solid lines in the top panel), which suggests that the concept of an interfacial layer indeed possesses significance.

The bottom panel of Fig. 1 shows experimental results obtained by Lu and Huang [25]. These researchers study the NaNO _3_/KNO _3_ (60:40) liquid base salt mixture doped with alumina nanoparticles of two distinct sizes. Their key observation is that *c*_*P*_ is larger for the nanofluid containing the larger nanoparticles under otherwise identical conditions. Using again Eq. 5, we find reasonable agreement with the experimental data when *λ* is around 2.

**Fig. 2:**
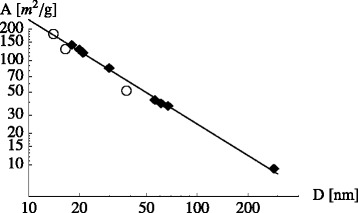
Specific surface area, *A*, vs. particle diameter, *D*. *Diamonds*: *N*_2_-BET data for carbon black from [40] (table 3.10 (p. 282); *circles*: *N*_2_-BET data for silica from [40] (table 3.19 (p. 300). The solid line is a *A*∝*D* ^−1^-fit to the carbon black data

But how is *λ* related to the interface? A standard technical specification available for nanoparticles is their surface area per mass unit, *A* (usually given in m ^2^/g). This surface is determined using different probe molecules, i.e., small ones like nitrogen or larger ones like CTAB. Typical values are on the order of 100 m ^2^/g. Actual values may deviate from this one by factors between 2 and 3. However, for the present discussion, this is not essential. Specifically, we can estimate the mass ratio *M*_*i*_/*M*_np_ via *M*_*i*_/*M*_np_≈*Aρ*_liq,*i*_*Δ*. Here *ρ*_liq,*i*_ is the average liquid density in the interfacial layer with thickness *Δ*. Furthermore, it is interesting to note that *A* is found to be inversely proportional to the particle radius *R* (cf. [40]) as shown in Fig. 2. Using this estimate *M*_*i*_/*M*_np_, Eq. 6 becomes

(7) $$ \lambda -1 \approx (1-\kappa) A \rho_{\text{liq},i} \Delta \;.   $$

What does this mean for the *λ* values used in the bottom panel of Fig. 1? Suppose we use *A*≈150 m ^2^/g for the small particles and *A*≈30 m ^2^/g for the large particles. In addition, we set *ρ*_liq,*i*_≈*ρ*_liq_≈1.8 g/cm ^3^ (cf. [25]). Of course, the particles induce structuring of the liquid close to their surface [41] and thus this may or may not be a good approximation. But it is fully sufficient if we want to explore the basic consequences of the interface. If we assume *Δ*=10 nm, then we need *κ*≈0.55 for the small particles and *κ*≈−0.30 for the large ones. Negative values for *κ* are in violation of thermal stability, however. We can avoid this by increasing the other quantities, i.e., *A*, *ρ*_liq_ or *Δ*. For instance, assuming *Δ*=100 nm, we obtain *κ*≈0.96 in case of the small particles and *κ*≈0.87 in the case of the large particles. Now the *κ* values are reasonable, but the width of the interface is unexpectedly large.

**Fig. 3:**
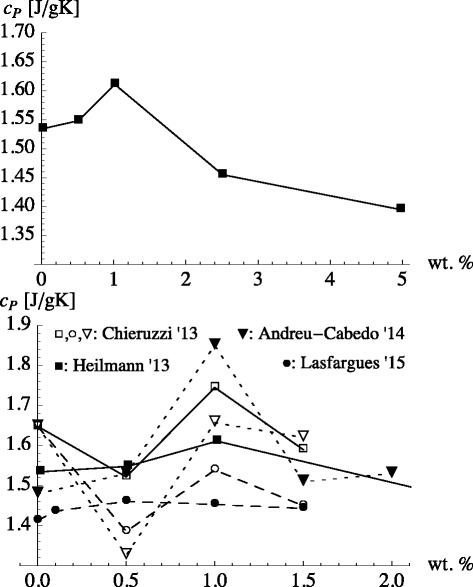
Specific heat capacity of the nanofluid vs. particle mass fraction. *Top*: Specific heat capacity of the eutectic base mixture NaNO _3_/KNO _3_ (60:40) to which Al _2_ *O*_3_ nanoparticles were added [33]. The temperature is 300 °C. *Bottom*: Specific heat capacity of the eutectic base mixture NaNO _3_/KNO _3_ (60:40). The *solid squares* again are Heilmann’s data [33]. *Open squares* connected via a *solid line* are data obtained by Chieruzzi et al. [30] for the same system. The *circles* (*triangles*) are data obtained for the eutectic base mixture NaNO _3_/KNO _3_ (60:40) to which TiO _2_ (SiO _2_) nanoparticles were added. Open symbols are data by Chieruzzi et al. [30]. *Solid circles* indicate TiO _2_ data by Lasfargues et al. [32]. *Solid triangles* indicate SiO _2_ data by Andreu-Cabedo et al. [34]

*Phenomenological heat capacity of nanofluids - the mesolayer concept*: The above discussion suggests that the nanoparticles induce a far reaching effect in the surrounding liquid. Of course, there is also the possibility that *κ* instead of *Δ* may become large, i.e. *c*_*P*,*i*_≫*c*_*P*,liq_. Even though large or diverging specific heat capacities may occur at a phase transition, i.e., in particular a second-order transition, these conditions are rather special and strongly temperature dependent [42]. Therefore, it is worth noting that most authors do observe only a slight change, usually a slight linear increase, of *c*_*P*_ in a fairly large temperature interval in the liquid state of the nanofluid (examples include Figs. 2 and 3 in [21], Fig. 3 in [23], Fig. 2 in [26], Figs. 2, 3, and 4 in [27], or Fig. 1 in [34]). All in all, a mechanism which increases *c*_*P*,*i*_ by a factor greater than two compared to the base fluid and at the same time is weakly dependent on temperature is difficult to imagine.

Next, we focus on recent work by Shin and Banerjee [23]. These authors find increases of the specific heat capacity of over 120 % relative to the base fluid (Li _2_CO_3_/K _2_CO_3_) (62:38). The nanoparticles are silica particles. The authors assume a *κ* of around 4. However, they do not provide a mechanism explaining this large increase of *c*_*P*,*i*_ relative to *c*_*P*,*liq*_. They also try to avoid large interface widths by proposing that the main effect is due to small nanoparticles with diameters of around 2 nm. This however makes it difficult to fix the attendant mass fraction (the overall *x*_np_ is around 1.5 wt.%). Small particles, as is illustrated in Fig. 2, do possess a large surface area. Thus, one may obtain a sufficiently large value for *λ*, according to Eq. 7, with a moderate *Δ*.

There is another subtlety worth mentioning. Apparently, the authors do observe segregation of their nanofluid into two “phases,” called types A and B, during preparation. The large increases of *c*_*P*_ reported in the paper are solely observed in the type A nanofluid, apparently containing well-dispersed nanoparticles <20 nm. However, it is not clear how large the mass content nanoparticle really is in type A. If we calculate the average center of mass separation for 2-nm particles at 1.5 wt.%, we obtain around 7 nm (on a cubic lattice). However, the transmission electron micrograph of a type A sample printed in the paper shows particles separated much farther than this. The picture is consistent with 20-nm particles as the dominant species, which yield an average separation of around 70 nm. For these particles, we obtain *Δ*≈50 nm if *κ*=4 is assumed. Because large *κ* values, and 4 already is large, are difficult to rationalize, this again suggest that *Δ* is in the 100-nm range. It is also worth mentioning that in a previous publication, the same authors [22], based on the same system (with a different silica supplier), do find a mere 20 % enhancement of *c*_*P*_. The reason for this substantial discrepancy is not clear.

Finally, we want to address the experimental observation of a maximum specific heat as a function of nanoparticle concentration. In Eq. 5, *c*_*P*_ depends linearly on *x*_np_. Therefore, this “theory” cannot describe results where *c*_*P*_ plotted vs. nanoparticle concentration is not monotonous. Figure 3 (top panel) shows measurements of Heilmann [33], which exhibit a rather distinct maximum at 1 wt.%. Unfortunately, if we include data by two other groups, i.e., Chieruzzi et al. [30] and Lasfargues et al. [32], matters become less clear as shown in Fig. 3. Open squares connected via a solid line are data obtained by Chieruzzi et al. [30] for the same system. We notice that while Chieruzzi et al. also obtain a maximum at 1 wt.%, their *c*_*p*_ of the plain salt mixture is significantly higher than Heilmann’s at roughly the same temperature. Thus, Chieruzzi et al. observe an initial decrease of *c*_*P*_. This is also true if they use TiO _2_ (open circles) or SiO _2_ (open down triangles) nanoparticles. Their maximum at 1 wt.% in the case of SiO _2_ is in rough accord with the data obtained by Andreu-Cabedo et al. [34] for the same system (solid down triangles). In the case of TiO _2_, Chieruzzi et al.’s results can be compared to the results obtained by Lasfargues et al. [32]. Aside from the discrepancy of the *c*_*P*_ values of the base salt, Lasfargues et al. find their *c*_*P*_ maximum at significantly lower concentration. The large scatter exhibited by *c*_*P*_ obtained for the pure base salt in the different studies (at about the same temperatures) illustrates that differences in the system preparation or (slight) differences in composition tend to obscure the overall picture.

*The interacting mesolayer model*: There is an explanation for the observed *c*_*P*_ maximum if we accept the above idea that the “interfacial layer” induced by a nanoparticle in the surrounding fluid is significantly wider than currently assumed. Notice that Eq. 5 does not account for the overlap between “interfacial layers” of nanoparticles. There is of course no need for this within the nanolayer concept, because at the nanoparticle concentrations of interest, no significant overlap of nanolayers occurs. If the layers are much wider, then overlap occurs even at very small nanoparticle concentrations, and this, as we shall see, can produce the *c*_*P*_ vs. *x*_np_ maximum.

We can extend Eq. 5 to include this idea as follows. The total mass of the entire system, *M*, is, as already pointed out, composed of three distinct contributions, i.e.,

(8) $$ M=M_{\text{liq},b} + M_{i} + M_{\text{np}} \;.  $$

Here, *M*_liq,*b*_ is the mass of the bulk liquid not affected by the presence of nanoparticles. *M*_*i*_ is the mass of the liquid affected by the nanoparticles, i.e., the mass of all interface layers combined. *M*_np_ is the mass of all nanoparticles combined. If we keep on adding nanoparticles, then we may reach a concentration when

(9) $$ M_{\text{liq},b}=0 \;.  $$

There simply is no bulk liquid left unaffected by the presence of nanoparticles. Beyond this concentration, Eq. 5 is no longer valid. It is replaced by the equation

(10) $$ c_{p}= \frac{M_{i} c_{P,i} +M_{\text{np}} c_{P,\text{np}}}{M}  $$

or

(11) $$ c_{p}= \kappa c_{P,\text{liq}} + x_{\text{np}} \left(c_{P,\text{np}} - \kappa c_{P,\text{liq}} \right)  $$

using the above terminology. The crossover mass fraction, $x_{\text {np}}^{\prime }$, beyond which Eq. 5 should be replaced by Eq. 11 according to Eq. 9 is

(12) $$ x_{\text{np}}^{\prime} = \frac{1}{1 + M_{i}/M_{\text{np}}} \;.  $$

**Fig. 4:**
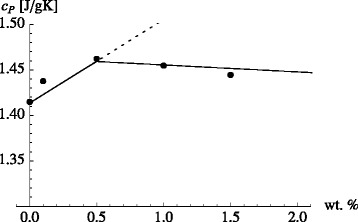
Interacting mesolayer model compared to the experiment. Equation 5 in conjunction with Eqs. 11 and 12, (*solid line*) in comparison to the data of Lasfargues et al. [32] from Fig. 3 (*bottom panel*). The *dotted line* is the continuation of Eq. 5 beyond $x_{\text {np}}^{\prime }$

In reality, there is no distinct $x_{\text {np}}^{\prime }$, because the interfacial shells influenced by the individual nanoparticles will begin to overlap gradually upon increasing the nanoparticle concentration. Nevertheless, we are interested in the basic consequences of the overlap for which this crude model is sufficient. Figure 4 shows the result of this new model, i.e., Eq. 5 in conjunction with Eqs. 11 and 12, (solid line) in comparison to the data of Lasfargues et al. [32] from Fig. 3 (bottom panel). We use the experimental values for *c*_*P*,liq_ and *c*_*p*,np_. *κ* and *M*_*i*_/*M*_np_ are adjustable parameters. Here, *κ*=1.035 and *M*_*i*_/*M*_np_=200. The required increase of *c*_*P*,*i*_ in comparison to *c*_*P*,liq_ is quite small (about 4 %). The width of the interfacial layer, *Δ*, however is about 4 times the diameter of the nanoparticles. We have assumed that the density of the bulk liquid is the same as the density of the liquid in the interface layer. Even though computer simulations have shown that solid surfaces may induce pronounced density variations in a liquid, the latter rarely extend beyond three to four molecular diameters away from the surface (e.g., [43]). Thus, the average density in the wide layer considered here remains close to the liquid bulk density.

**Fig. 5:**
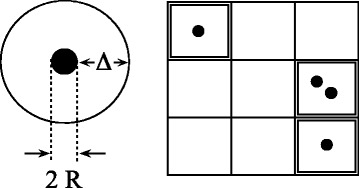
Cell model. *Left*: A nanoparticle of radius *R* surrounded by its interface layer of thickness *Δ*. *Right*: Cubic lattice model of a nanofluid. Cells containing a box with a particle at their center are occupied by a nanoparticle including its interface layer. Cells may by occupied multiple times. Depicted is a single double occupied cell. Empty cells contain bulk base fluid, i.e., base fluid not affected by the presence of nanoparticles

We can refine this model and combine Eq. 5 with Eqs. 11 and 12 into a single expression. The underlying idea is depicted in Fig. 5. The sketch on the right shows a lattice model description of the nanofluid. Cells containing a box with a particle at their center are occupied by a nanoparticle including its interface layer. Cells may be occupied multiple times. Depicted is a single double occupied cell. Empty cells contain bulk base fluid, i.e., base fluid not affected by the presence of nanoparticles. Notice that in this model, there is no partial overlap. Interfacial layers overlap completely or not at all.

Assuming that the nanoparticles are uncorrelated, we may calculate the average volume base fluid, which is not affected by the presence of nanoparticles. Referring to the sketch, this is the average number of empty cells, *n*_*e*_, on a cubic lattice possessing *n*_*o*_ cells into which *n* nanoparticles are placed randomly, i.e.,

(13) $$ n_{e} = n_{o} \exp [-n/n_{o}] \;.  $$

Here, we also assume that *n*_*o*_ and *n* are large. Assuming that the liquid density inside the interface layer is the same (or nearly the same) as outside, we use the average liquid density $\bar \rho = \rho _{\text {np}} x_{\text {np}} + \rho _{\text {liq}} (1-x_{\text {np}})$ to rewrite Eq. 13 into

(14) $$ x_{\text{liq}}^{\prime} = \frac{\rho_{\text{liq}}}{\bar \rho} Y  $$

with

(15) $$ Y= \exp \left[x_{\text{np}} \left(x_{\text{np}} + \frac{\rho_{\text{liq}}}{\rho_{\text{np}}} \left(1-x_{\text{np}}\right) \left(1+\frac{\Delta}{R}\right)^{3} \right)\right]\,.  $$

The quantity $x_{\text {liq}}^{\prime }$ is the mass fraction of bulk liquid, i.e., base liquid unaffected by the presence of nanoparticles. The specific heat capacity in this model is

(16) $$ c_{P} = \left[\kappa (1-x_{\text{np}}) + (1-\kappa) x_{\text{liq}}^{\prime} \right] c_{p,\text{liq}} + x_{\text{np}} c_{P,\text{np}}\,.  $$

**Fig. 6:**
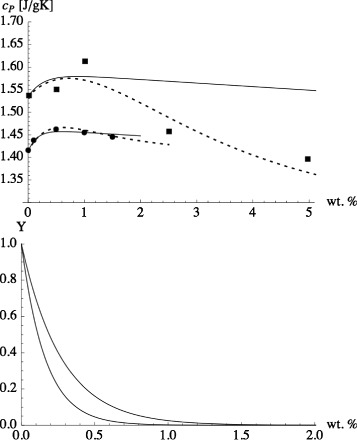
Refined interacting mesolayer model compared to the experiment. *Top*: Equation 16 (*solid lines*) compared to the experiments of Lasfargues et al. [32] (*solid circles*) and Heilmann [33] (*solid squares*) from Fig. 3 (*bottom panel*). We use the experimental liquid densities of the respective source - even though the base salt is the same in both cases. Here, *κ*=1.035 as in Fig. 4. *Δ*/*R*=10 in the case of the Lasfargues et al. data and *Δ*/*R*=8 in the case of the Heilmann data. In addition, we use *ρ*_liquid_=1.8 g/cm ^3^, $\rho _{\text {TiO}{_{2}}} =3.9$ g/cm ^3^, *ρ*_alumina_=3.6 g/cm ^3^, and *c*_*P*,np_ between 0.7 and 0.8 J/(gK). *Dashed lines*: Variable *κ* as explained in the text. Bottom: *Y* vs. weight percent corresponding to the two *solid lines* above. The *lower (upper) curve* corresponds to the fit of the Lasfargues (Heilmann) data

A comparison of this formula to experimental data is shown in Fig. 6. The expression works quite well in the case of the Lasfargues et al.’s data, whereas Heilmann’s data are in mere qualitative agreement.

Improved agreement is achieved upon introducing a concentration independent *κ*. When the interfacial layers of two nanoparticles overlap, *κ* may be different in the overlap region in comparison to the part of the interface layer which is not overlapping. The dashed curves in Fig. 6 are obtained with a variable *κ*, which is given by

(17) $$ \kappa = \kappa_{\text{max}} Y - \kappa_{\text{min}} (1-Y) \;.  $$

Notice that *κ*=*κ*_max_ when *Y*=1, which means that little or no overlap between shells occurs on average. *κ*=*κ*_min_ when *Y*=0. This means that the overlap is at a maximum. We use *κ*_max_=1.2(1.14), *κ*_min_=0.87(1.02), and *Δ*/*R*=3.5(5.5) for the Heilmann (Lasfargues et al.) data. The theory now is in better agreement with both data sets. Notice that *κ* is reduced with increasing overlap.

But why should *κ* be reduced (below one) with increasing overlap? After all, our phenomenological model matches the experiments showing enhanced heat capacity only if *κ*_max_ is larger than one. The presence of a nanoparticle may apparently enhance the specific heat capacity in a surrounding liquid shell of width *Δ* by a factor *κ*_max_>1. So why should the presence of a second nanaoparticle in the proximity of the first have the opposite effect? The interpretation is that increasing overlap means that nanoparticles may be getting close. This imposes significant positional ordering on the liquid molecules, which may form a “solid-like” layer structure between the two “walls.” This type of surface-induced ordering can for instance be measured using the surface force apparatus (e.g., [44]). Experiments show that *c*_*P*,solid_ of the base salt in its solid state is significantly lower than the corresponding *c*_*P*,liq_. An example is [26], where the authors provide both *c*_*P*,solid_ and *c*_*P*,liq_. Outside the immediate transition region, both specific heat capacities are well described by a linear dependence on temperature. If we linearly extrapolate *c*_*P*,solid_ into the temperature regime where *c*_*P*,liq_ is measured, then *c*_*P*,solid_ is up to 10 % less than *c*_*P*,liq_. Thus, if the *c*_*p*_ of the base liquid in this overlap area between nanoparticles becomes more like *c*_*P*,solid_, we should expect *κ* to decrease - even below one. Due to the complexity of the systems, we believe that this point should be studied using molecular simulation techniques rather than analytical approaches. The foremost question however remains - why is *κ*_max_ larger than one in the first place? We shall return to this question below.

**Fig. 7:**
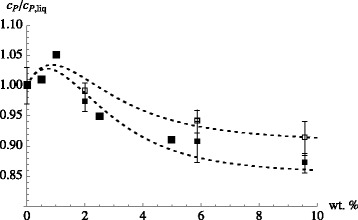
Refined interacting mesolayer model with variable *κ* compared to the experiment. *c*_*P*_/*c*_*P*,liq_ vs. weight percent nanoparticle. Comparison of the interacting mesolayer model with variable *κ* to experimental data. The larger *solid squares* are the Heilmann data (taken at 300 °C) from the previous figure. The smaller symbols including *error bars* are the data points shown in the *bottom panel* of Fig. 1 (based on averages in the temperature range from 290 to 335 °C). The lower of the two *dashed lines* agrees with the dashed line in the previous figure. The upper one, for the larger particles, is obtained by increasing *κ*_min_ from 0.87 to 0.93

When we discussed Fig. 1 (bottom panel) in the context of Eq. 5, we did reach the conclusion that *κ*<1 was necessary to explain the data. In hindsight, we can now say that the decrease of *c*_*P*_ in the bottom panel of Fig. 1 as *ϕ* is increased is observed because the data, in the range 0.02<*x*_np_<0.1, lie beyond the maximum of *c*_*P*_ for this nanofluid. This point is shown more clearly in Fig. 7. The figure combines the Heilmann data from Fig. 6 with the data from the bottom panel of Fig. 1. One of the dashed lines is again the upper dashed line from Fig. 6, i.e., the result of our recent theory with a variable *κ* parameter. In order to capture the particle size effect discussed in the context of Fig. 1 (bottom panel), a second curve with a slightly increased *κ*_min_ is needed. Notice the closeness of these values for *κ*_min_ to the *κ* values already conjectured during the previous discussion of the bottom panel of Fig. 1.

We note that we did not alter the ratio *Δ*/*R* according to the two very different mean particle sizes used by Lu and Huang [25]. The ratio *Δ*/*R* determines the position of the *c*_*P*_ maximum in terms of nanoparticle concentration. We do not think that *Δ* and *R* are independent. The effect of nanoparticles on viscosity or, in the case of elastomers, the effect of nanoparticles on the elastic modulus is derived from hydrodynamic [45–47] or elastic equations [48]. The only length scale in these theories is the particle radius, *R*. The effect of the presence of a particle in the surrounding medium at a distance *r* from the particle center decays algebraically in terms of certain powers of *R*/*r*. We consider it very likely that the effect of the particles on *c*_*P*_ will follow from an analogous mathematical description. This means that *Δ* has no precise meaning. It should rather be thought of as some *r* at which *R*/*r* has decayed below a certain value. In this sense, we view *Δ*/*R* as one single parameter - independent of the actual particle radius.

However, we still need to tackle the question what possible mechanism leads to *κ*_max_>1. Before addressing this point, we mention two observations, based on experimental evidence, which may provide helpful hints. First, it is worth noting that experimental studies find that the qualitative effect of nanoparticles on *c*_*P*_ appears to be the same in the solid and in the liquid state of the respective nanofluids (e.g., [21, 25–27, 35]). The solid state being of course not so much of technical importance. In the aforementioned studies, the solid state *c*_*P*_ rises close to linearly with increasing temperature in a rather wide range of temperatures. In most of these studies (exceptions are [26], where *c*_*P*_ is close to constant in the studied temperature range, and [35], where the authors find a decrease of *c*_*P*_ with rising temperature), this holds true also in the liquid state. These similarities between solid and liquid phase are interesting, because the solid state theory of specific heat capacity is much better developed than its counterpart for liquids. Secondly, we note that base salt mixtures like NaNO _3_/KNO _3_ (60:40) or the Li _2_CO_3_/K _2_CO_3_ eutectic (as well as water) possess a *c*_*P*_, which is rather close to *c*_*V*_ calculated using the Dulong-Petit law.

One recent approach to the heat capacity in liquids, which explicitly exploits similarities between liquids and solids, is the phonon theory by Bolmatov et al. [49]. Bolmatov et al. show that their theory yields the isochoric heat capacity, *C*_*V*_, vs. *T* for a great number of fluids in quite good quantitative agreement with experimental measurements. The key idea, based on an old proposition of Frenkel, is that a liquid becomes solid-like at frequencies larger than *τ*^−1^∼*η*^−1^, when it may support shear waves. Here, *τ* is a certain relaxation time and *η* is the liquid’s viscosity. The authors derive an expression for the internal energy of the liquid consisting of contributions due to longitudinal phonons as well as transverse (shear) phonons beyond *τ*^−1^ and a diffusion contribution below *τ*^−1^. We emphasize this theory also because of one particular aspect. Since Einstein [45, 46], it is well known how the addition of dispersed particles to a liquid increases its viscosity, i.e., *η*=*η*^∗^(1+2.5*ϕ*) for *ϕ*≪1 (cf. [3]). Here, *η*^∗^ is the viscosity of the pure liquid and *ϕ* is the volume fraction of the added particles. In the present case, this means that *τ*^−1^ should decrease, i.e., the nanoliquid is able to support more shear modes at a particular temperature in comparison to the base fluid. This in turn increases *C*_*V*_, and thus addition of nanoparticles, at first glance, leads to an increase of the isochoric heat capacity. However, the full relation between *τ*^−1^ and *η*^−1^ in [49] is *τ*^−1^=*G*_*∞*_/*η*, where *G*_*∞*_ is the infinite-frequency shear modulus. Whether or not *G*_*∞*_ possesses the same dependence on the concentration of added particles as *η* (cf. [48]) is a subtle question. If it does, then there is no effect of the nanoparticles on *τ*^−1^. In the case of filled rubbers, which have much in common with liquids, it is well known that the glass transition is indeed not shifted by addition of nanoparticles (even at large amounts), i.e., the attendant relaxation times are not dependent on filler.

Even if the approach of Bolmatov et al. does not provide an immediate explanation of the enhanced *c*_*P*_ in nanofluids, we stick with the phonon idea and peruse it from another angle. The following is a simple example, illustrating how a small perturbation on the structure of the liquid, within the phonon picture of Bolmatov et al., may yield an increase of the heat capacity. Consider a one-dimensional perturbed, i.e., non-linear, oscillator, possessing the Hamiltonian

(18) $$ \mathcal{H} =\hbar \omega \left(- \frac{1}{2} {\partial_{q}^{2}} + \frac{1}{2} q^{2} - \lambda q^{4}\right) \,.  $$

**Fig. 8:**
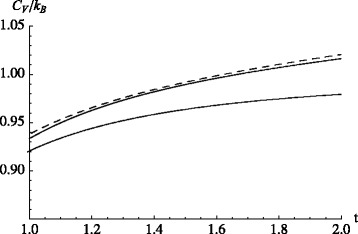
Heat capacity of a one-dimensional perturbed oscillator. *C*_*V*_/*k*_*B*_ vs. reduced temperature $t=k_{B} T /(\hbar \omega)$. The *lower (upper) solid line* is the result for the harmonic (perturbed) oscillator, i.e., *λ*=0(0.01). The *dashed line* is the numerical solution of the perturbed oscillator’s heat capacity

Here, *λ* is a small parameter. First-order perturbation theory yields the energy eigenvalues $E_{n} = \hbar \omega (n + 1/2 + (3/2) \lambda (n^{2} + n +1/2))$. Notice that a *q*^3^ perturbation does not contribute in the first order, and this is why a *q*^4^ perturbation is considered here instead. We obtain *C*_*V*_ via *C*_*V*_=*∂*〈*E*〉/*∂T*|_*V*_ and $\langle E \rangle =\partial _{(-\beta)} \ln \sum _{n=0}^{\infty } \exp [-\beta E_{n}]$, where *β*=(*k*_*B*_*T*)^−1^. Assuming that $\beta \hbar \omega \lambda \ll 1$, we use the expansion $\exp [-\beta E_{n}] \approx (1-\hbar \omega (3/2) \beta \lambda (n^{2} + n +1/2)) \exp [-\beta \hbar \omega (n+1/2)]$, which yields

(19) $$ C_{V}/k_{B} \approx 1 + 6 \lambda t - 1/\left(12 t^{2}\right)  $$

in leading order of (small) *λ* and (large) $t=k_{B} T / (\hbar \omega)$. Had we included a term *χq*^3^ in $\mathcal {H}$, then its leading order contribution to *C*_*V*_ would have been 15*χ*^2^*t*. Figure 8 compares Eq. 19 to the exact *C*_*V*_ of the harmonic oscillator (*λ*=0) as well as to the numerical solution of the perturbed oscillator according to Eq. 19. The figure illustrates that even a small perturbation (here, *λ*=0.01) yields a significant increase of *C*_*V*_ at high temperatures. Note that when *λ* is positive, the perturbation increases the width of the oscillator potential, which reduces the spacing between eigenvalues and thus increases *C*_*V*_. What we propose is in essence that the nanoparticles induce a long-range perturbation into the surrounding liquid (or solid in the case of the solid phase), which gives rise to enhanced anharmonicity.

Notice that anharmonic molecular interactions are the source of thermal expansion. Both heat capacity and thermal expansion are closely related - at least in the case of solids (e.g., *§* 67 in [50]; To the best of the author‘s knowledge, this particular relation has not been explored thus far in experiments on nanofluids in the present context. Thermal expansion of water-based nanofluids has been obtained in several studies primarily aimed at quantities other than heat capacity (e.g., [51]). But in these water-based systems, excess effects due to the presence of nanoparticles are difficult to discern.) Because the expression Bolmatov et al. derive for *C*_*V*_, based on their phonon theory, also contains the thermal expansion coefficient (in fact, their *C*_*V*_ increases with increasing thermal expansion), the calculation or modeling of the latter may offer a theoretical approach to *c*_*P*_. At least from the perspective of molecular computer simulation, thermal expansion appears to be the simpler quantity.

We remark in closing that recently, Tiznobaik, Shin, and Banerjee [28, 29] have carried out an interesting experiment, which shows that addition of NaOH in minuscule amounts prevents the formation of structure and eliminates the previously observed *c*_*P*_ enhancement. In the present picture, we would interpret the effect of NaOH as one that relaxes anharmonicity effects.

## Conclusions

Specific heat enhancements of the type observed thus far requires one or both of the following to be true: (i) Every particle is surrounded by a nanolayer with a *c*_*P*_ exceeding the bulk fluid’s *c*_*P*_ by a large factor. This is necessary to explain the large increases observed in some experiments in which *c*_*P*_ is enhanced through addition of minute amounts of nanoparticle to the base fluid. (ii) The effect of the particles on the base fluid has a long range (100 nm or more), which only requires a moderate change of *c*_*P*_ in the attendant mesolayer. The new phenomenological theory, i.e., the interacting mesolayer model, developed on the assumption of (ii) can explain the occurrence of the *c*_*P*_ vs *x*_*np*_ maximum. It contains three parameters: (1) *Δ*/*R*, i.e., the width of the mesolayer in units of *R*, the particle radius; (2) *κ*_max_, the ratio of the average specific heat capacity inside the mesolayer at vanishing overlap to the specific heat capacity of the base fluid at the same temperature; (3) *κ*_min_, the ratio of the average specific heat capacity inside the mesolayer at maximum overlap to the specific heat capacity of the base fluid at the same temperature. Thus far, no microscopic theory exists, from which these parameters can be computed. Nevertheless, we have proposed a physical picture according to which *κ*_max_>1 is possible, based on induced enhanced anharmonicity of the molecular interactions, as well as a mechanism for the reduction of *κ* to *κ*_min_, via ‘solidification’ of the base liquid due to confinement.

## Abbreviations

BET: Brunauer-Emmett-Teller
CTAB: dodecyl-trimethyl-ammonium
CNT: carbon nanotubes
SDS: sodium-dodecyl-sulfate
TEM: transmission electron micrograph
